# A risk score based on polyamine metabolism and chemotherapy‐related genes predicts prognosis and immune cells infiltration of lung adenocarcinoma

**DOI:** 10.1111/jcmm.18387

**Published:** 2024-06-26

**Authors:** Minjun Du, Xiangzhi Meng, Boxuan Zhou, Weijian Song, Jianwei Shi, Mei Liang, Yicheng Liang, Yushun Gao

**Affiliations:** ^1^ Department of Thoracic Surgery, National Cancer Center/National Clinical Research Center for Cancer/Cancer Hospital Chinese Academy of Medical Sciences and Peking Union Medical College Beijing China; ^2^ Department of thoracic surgery, Sun Yat‐sen Memorial Hospital Sun Yat‐sen University Guangzhou China

**Keywords:** immune infiltration, lung adenocarcinoma, platinum‐based chemotherapy, polyamine metabolism, prognosis

## Abstract

We aimed to explore whether the genes associated with both platinum‐based therapy and polyamine metabolism could predict the prognosis of LUAD. We searched for the differential expression genes (DEGs) associated with platinum‐based therapy, then we interacted them with polyamine metabolism‐related genes to obtain hub genes. Subsequently, we analysed the main immune cell populations in LUAD using the scRNA‐seq data, and evaluated the activity of polyamine metabolism of different cell subpopulations. The DEGs between high and low activity groups were screened to identify key DEGs to establish prognostic risk score model. We further elucidated the landscape of immune cells, mutation and drug sensitivity analysis in different risk groups. Finally, we got 10 hub genes associated with both platinum‐based chemotherapy and polyamine metabolism, and found that these hub genes mainly affected signalling transduction pathways. B cells and mast cells with highest polyamine metabolism activity, while NK cells were found with lowest polyamine metabolism activity based on scRNA‐seq data. DEGs between high and low polyamine metabolism activity groups were identified, then 6 key genes were screened out to build risk score, which showed a good predictive power. The risk score showed a universal negative correlation with immunotherapy checkpoint genes and the cytotoxic T cells infiltration. The mutation rates of EGFR in low‐risk group was significantly higher than that of high‐risk group. In conclusion, we developed a risk score based on key genes associated with platinum‐based therapy and polyamine metabolism, which provide a new perspective for prognosis prediction of LUAD.

## 1 INTRODUCTION

Lung adenocarcinoma (LUAD), a prevalent subtype of lung cancer, has responsible for nearly 40% of all lung cancer cases.^1^, ^2^ Despite notable advancements in the treatment strategy of LUAD, the prognosis did not improve significantly in recent years owing to its resistance to chemotherapy.^3^, ^4^, ^5^ In LUAD, platinum combined with other drugs is still the main treatment regimen, with significant effects in early stage. With the progression of LUAD, the resistance of tumour cells to platinum is an important factor affecting the efficacy of chemotherapy and prognosis of LUAD.^6^ Consequently, it is important to develop prediction tools that can predict the prognosis of LUAD patients received platinum‐based chemotherapy.

The polyamines, including putrescine, spermidine and spermine, are polycationic alkylamines at low concentrations in mammalian cells.^7^ These molecules play a crucial role in various cellular processes, such as protein and nucleic acid synthesis,^8^ and regulation of cell‐to‐cell communication.^9^ Normal cell growth relies on adequate levels of polyamines, and their depletion leads to cell growth arrest. In cancer, polyamine metabolism is often dysregulated, and increased polyamine levels are necessary for tumour transformation and progression.^10^ Study based on metabolomics and proteomics have suggested that polyamine biosynthesis was elevated in LUAD.^11^ However, there are few studies about the relationship between polyamine metabolism and prognosis of LUAD.

In our study, we investigated whether the genes associated with both platinum‐based therapy and polyamine metabolism could be used to predict the prognosis of LUAD. Through screening, we identified six genes (RORA, CLEC2D, NPC2, A2M, FYN and KLF4) with prognostic significance, then they were utilized to construct a risk score model. We successfully validated the efficacy of our model in both the training and external validation cohorts and further characterized the immune and mutation landscape between two risk groups in the LUAD.

## 2 METHODS

### 2.1 Data acquisition

To compare for the differential expression genes (DEGs) related with platinum‐based therapy, 62 LUAD patients who did not receive adjuvant therapy in the GSE14814 cohort were included, and 95 patients received cisplatin and vinorelbine in GSE108492 cohort were also enrolled. The scRNA‐seq data from 10 LUAD patients in the GSE131907 dataset was used for the analysis of tumour microenvironment. 503 LUAD patients in the TCGA‐LUAD cohort with corresponding clinical information were enrolled as training cohort. 85 patients with LUAD bulk RNA‐seq data were enrolled from the GSE30219 cohort as validation cohort.

### 2.2 Procurement of genes associated with polyamine metabolism

Fifteen genes related to polyamine metabolism were obtained from the MSigDB database (https://www.gsea‐msigdb.org/gsea/index.jsp).

### 2.3 Differential expression analysis of bulk‐RNA seqcencing

DEGs related to platinum‐based therapy were selected by “limma” package. We performed differential gene analysis on the GSE108492 dataset and the GSE14814 dataset, which included 95 patients who received chemotherapy and 62 patients who did not. The criteria for DEGs selection were adjusted *p*‐value <0.05 and absolute log2 fold change (log2FC) >0.585. Then, the DEGs were intersected with the 15 polyamine metabolism‐related genes to obtain the hub genes associated with both platinum‐based chemotherapy and polyamine metabolism.

### 2.4 Functional enrichment analysis

Gene Ontology (GO) analysis and KEGG analysis of the identified DEGs were performed. The functional enrichment analysis was conducted using the R package “clusterProfiler” (version 4.0.5) on the GSE108492 dataset and the GSE14814 dataset. *p* < 0.05 was used to determine significant enrichment.

### 2.5 Single‐cell sequencing analysis

First, analysis was performed on scRNA‐seq data of the 10 LUAD samples from GSE131907. Cells with nFeature_RNA <9000 and percent.mt <25 were retained for further analysis. Dimensionality reduction clustering was implemented using SingleR annotation, yielding a total of 8 cell clusters: B cells, myeloid cells, endothelial cells, NK cells, epithelial cells, fibroblasts, mast cells and T cells. The tumour‐related pathway scores of the 8 cell clusters were calculated using PROGENy analysis. GSVA were used to calculate the activity of 50 tumour associated pathways for the 8 cell clusters. The AddModuleScore function of the Seruat package was used to obtain the scores of the 10 selected DEGs. Then, the FindAllmarkers function of the Seruat package was used to screen for DEGs between high score group and low score group, with an adjusted *p*‐value <0.05 and an absolute logFC >0.585.

### 2.6 Construction of risk score

Univariate Cox regression analysis was performed on DEGs derived from different groups of scRNA‐seq. Then, by using the Lasso regression machine learning algorithm, the TCGA‐LUAD cohort was used to construct a prognostic risk score model.

### 2.7 Immune cells and immune regulating cells infiltration analysis

Two methods were used to calculate immune infiltration score: ssGSEA and xCell algorithms. Box plots, heatmaps and scatter plots were used for visualization. ssGSEA was used to calculate the gene‐set enrichment score of individual samples to assess the levels of immune cell infiltration. xCell was used to quantify the abundance of immune cells and immune response‐related cells, based on transcriptome data.

### 2.8 Mutation and drug sensitivity analysis

Mutation profiles of the TCGA‐LUAD cohort were visualized. The “maftools” R package (version 2.12.0) was used to visualize mutation data for Low‐risk and High‐risk groups of risk score. Drug sensitivity scores was calculated for two risk groups using the “oncoPredict” R package by GDSC database.

### 2.9 Statistical analysis

All statistical analyses were performed using the R language. The univariable and multivariable Cox regression analysis was conducted using the “survival” and “survminer” packages. The student's *t*‐test was used to determine if there is a significant relationship between two variables. Pearson correlation analysis was used to assess the strength of the linear association between two variables. For all statistical analyses, *p*‐value <0.05 was considered statistically significant.

## 3 RESULTS

### 3.1 DEGs analysis of LUAD patients receiving and not receiving platinum‐based chemotherapy

**FIGURE 1:**
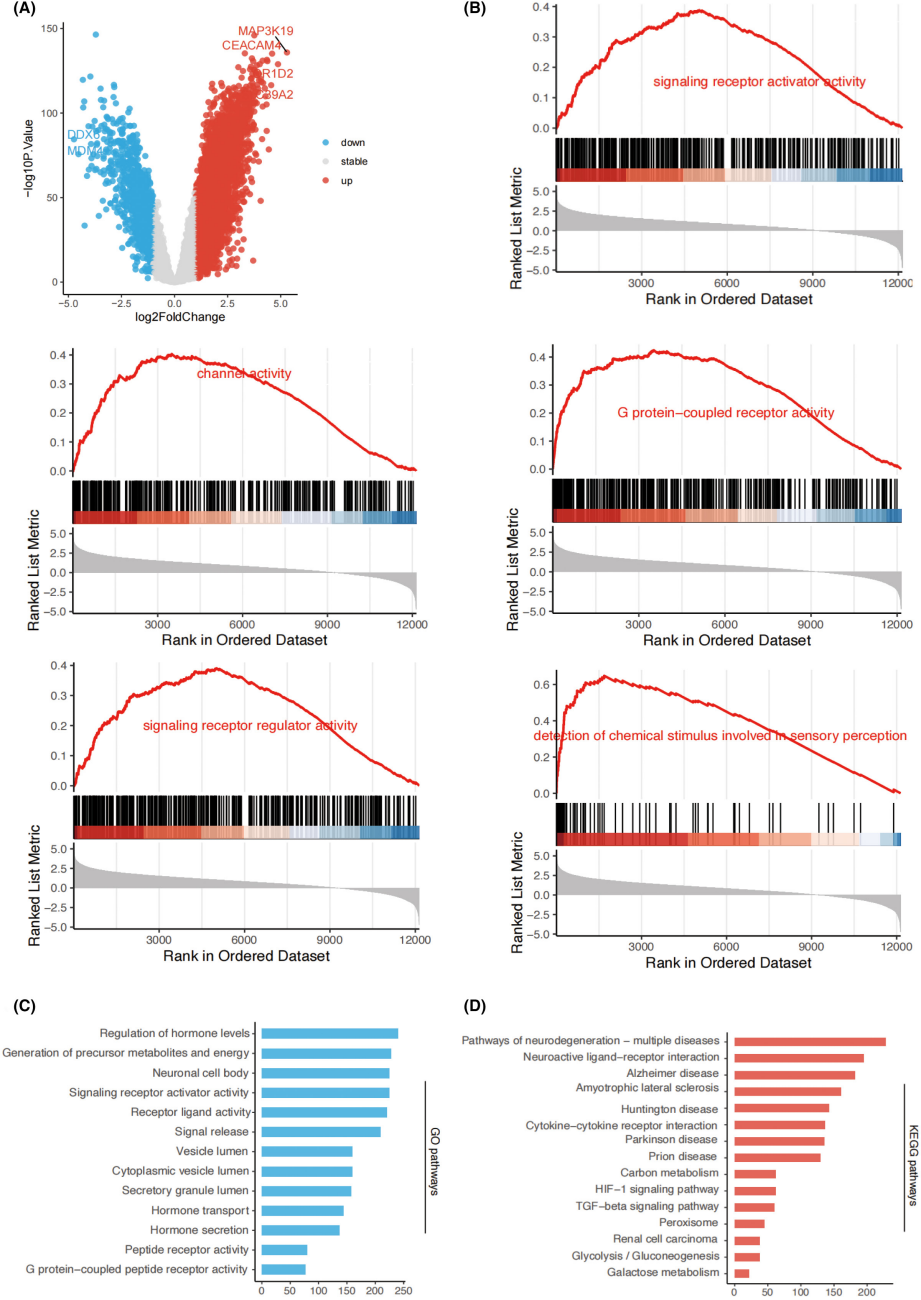
The pathway enrichment analysis of 10 hub genes. (A) DEGs related to platinum‐based chemotherapy. (B) GSEA derived GO enrichment of hub genes. (C) GO enrichment of hub genes. (D) KEGG enrichment of hub genes.

In order to fully explore the underlying mechanisms of the platinum‐based chemotherapy in LUAD, we searched for the DEGs between patients with platinum‐based chemotherapy (GSE108492) or without platinum‐based chemotherapy (GSE14814), and we got 362 DEGs (Figure 1A). Then, the DEGs were intersected with the 15 polyamine metabolism‐related genes, and we obtained 10 hub genes associated with both platinum‐based chemotherapy and polyamine metabolism. Based on GSEA analysis, we found the hub genes significantly associated with signalling receptor activator activity, G protein‐coupled receptor activity, channel activity, signalling receptor regulator activity and detection of chemical stimulus involved in sensory perception, suggesting signalling transduction pathways might play pivotal roles in regulating platinum‐based chemotherapy (Figure 1B). We further performed the KEGG and GO functional enrichment analysis, and found that signalling receptor activator activity, receptor ligand activity, HIF‐1 signalling pathway and TGF‐beta signalling pathway were highly enriched, which were similar with the results of GSEA analysis (Figure 1C,D).

### 3.2 Characterization and cell communication of cell subpopulations in LUAD

**FIGURE 2:**
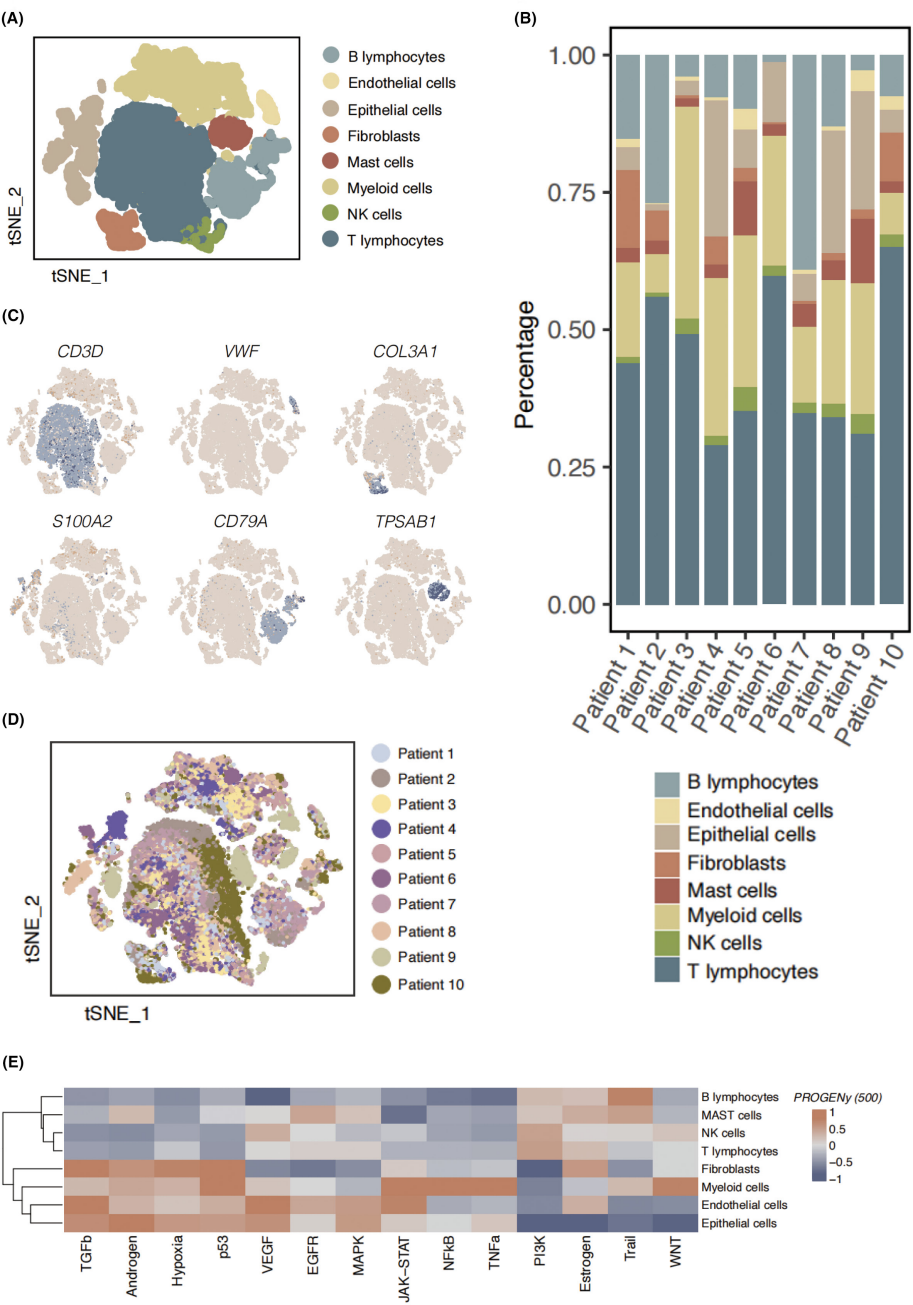
Characterization of cell subpopulations in LUAD. (A) The clustering of 8 cell populations, namely B lymphocytes, endothelial cells, epithelial cells, fibroblasts, mast cells, myeloid cells, NK cells, T cells. (B) The proportions of different cell populations in 10 LUAD patients. (C) The expression levels of typical marker genes, including CD3D, VWF, COL3A1, S100A2, CD79A, TPSAB1, in 10 LUAD patients. (D) The cell origin from different LUAD patients. (E) The PROGENy pathway enrichment of different immune cell types.

We analysed the main immune cell subpopulations in LUAD using the scRNA‐seq data. We identified 8 cell subpopulations (Figure 2A), namely B lymphocytes, endothelial cells, epithelial cells, fibroblasts, mast cells, myeloid cells, NK cells and T cells. We observed highest level of T cell infiltration across the 10 LUAD patients, suggesting a critical role of T cells in the immune microenvironment in LUAD. The proportion of epithelial cells ranked secondly, a majority of which could be identified as malignant cells (Figure 2B). The typical marker genes for annotation of the immune cell population were displayed in the Figure 2C. Of note, we annotated the T cells with CD3D, endothelial cells with VWF, mast cells with TPSAB, and B cells with CD79A. The cell origin from different LUAD patients were displayed in Figure 2D. The PROGENy pathway enrichment showed marked enrichment of classic cancer‐related signalling pathways in 8 cell subpopulations (Figure 2E), suggesting unique functional patterns of the LUAD‐infiltrating stromal cells and immune cells.

**FIGURE 3:**
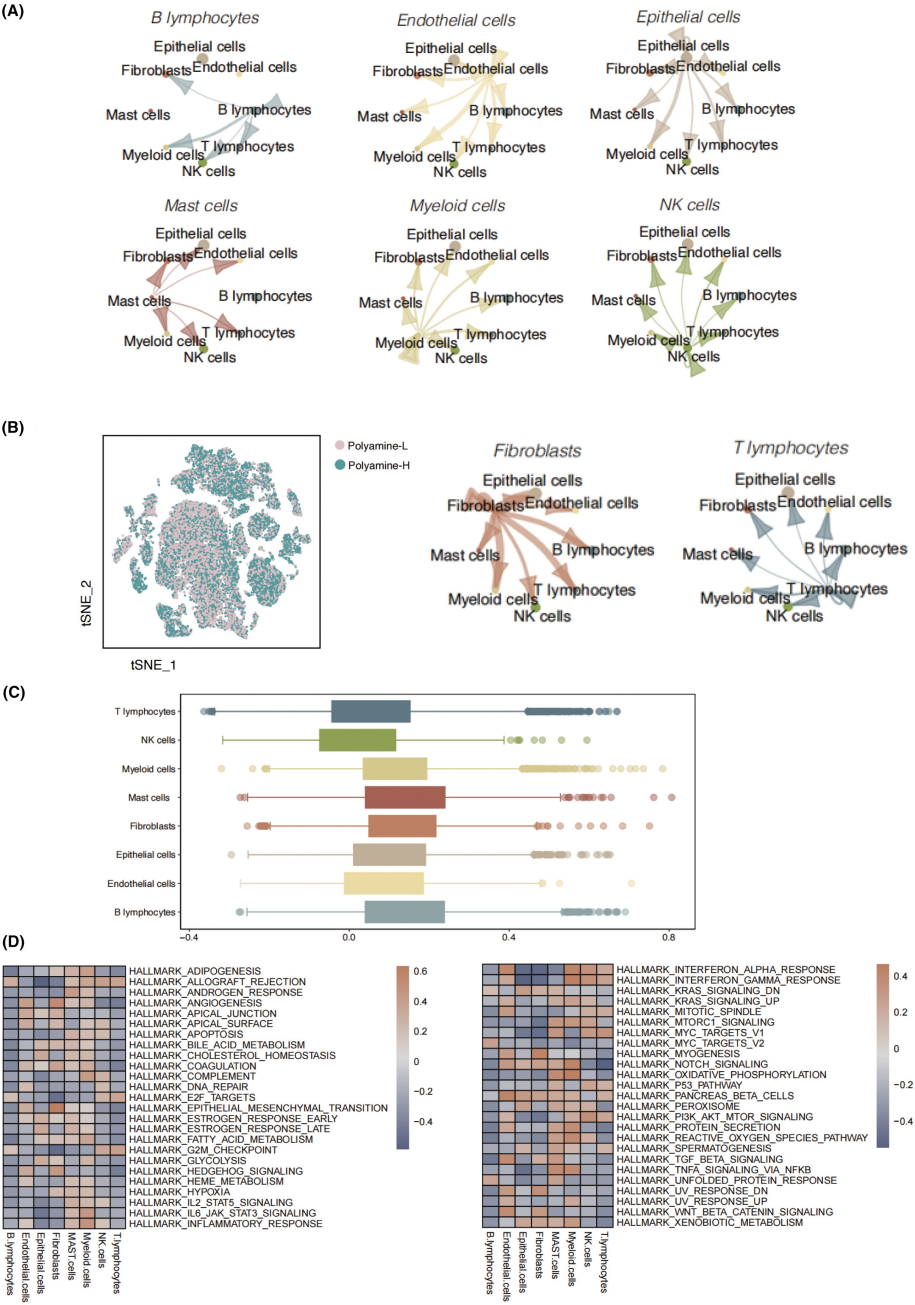
Cell communication of cell subpopulations and characterization of polyamine metabolism in LUAD. (A) Cell communication interactions among the eight cell populations. (B) The activities of high polyamine metabolism and low polyamine metabolism groups in LUAD. (C) The expression levels of polyamine metabolism related genes in LUAD, categorized by different cell populations. (D) The GSVA scores of different cell populations based on hallmark pathways.

We further investigated the cell communication pattern between 8 different cell types. We identified fibroblasts as the predominant cell type sending outgoing signals to all 7 other cell populations, especially the epithelial cells (Figure 3A). A heated cell‐to‐cell communication was found between the myeloid cells and endothelial cells.

### 3.3 Characterization of polyamine metabolism in LUAD

The tSNE diagram showed the activities of polyamine metabolism in LUAD by assessing the expressions of 10 hub genes (Figure 3B). We found that B cells and mast cells with highest activity of polyamine metabolism genes, followed by the fibroblasts, while NK cells were found with lowest polyamine metabolism activity (Figure 3C). DEGs were identified by distinguished the expression data between high polyamine metabolism activity group and low polyamine metabolism activity group, a total of 125 DEGs, which were subsequently subjected to the GSVA scoring analysis of different cell populations based on hallmark pathways. We took a special focus on the fibroblasts, which was highly enriched in the epithelial mesenchymal transition. Moreover, angiogenesis, TGF‐beta and WNT‐beta catenin signalling were prominently elevated in the fibroblasts, indicating a versatile role of fibroblasts in the LUAD (Figure 3D).

### 3.4 The establishment of a prognostic risk score model based on DEGs between polyamine metabolism and platinum‐based chemotherapy

**FIGURE 4:**
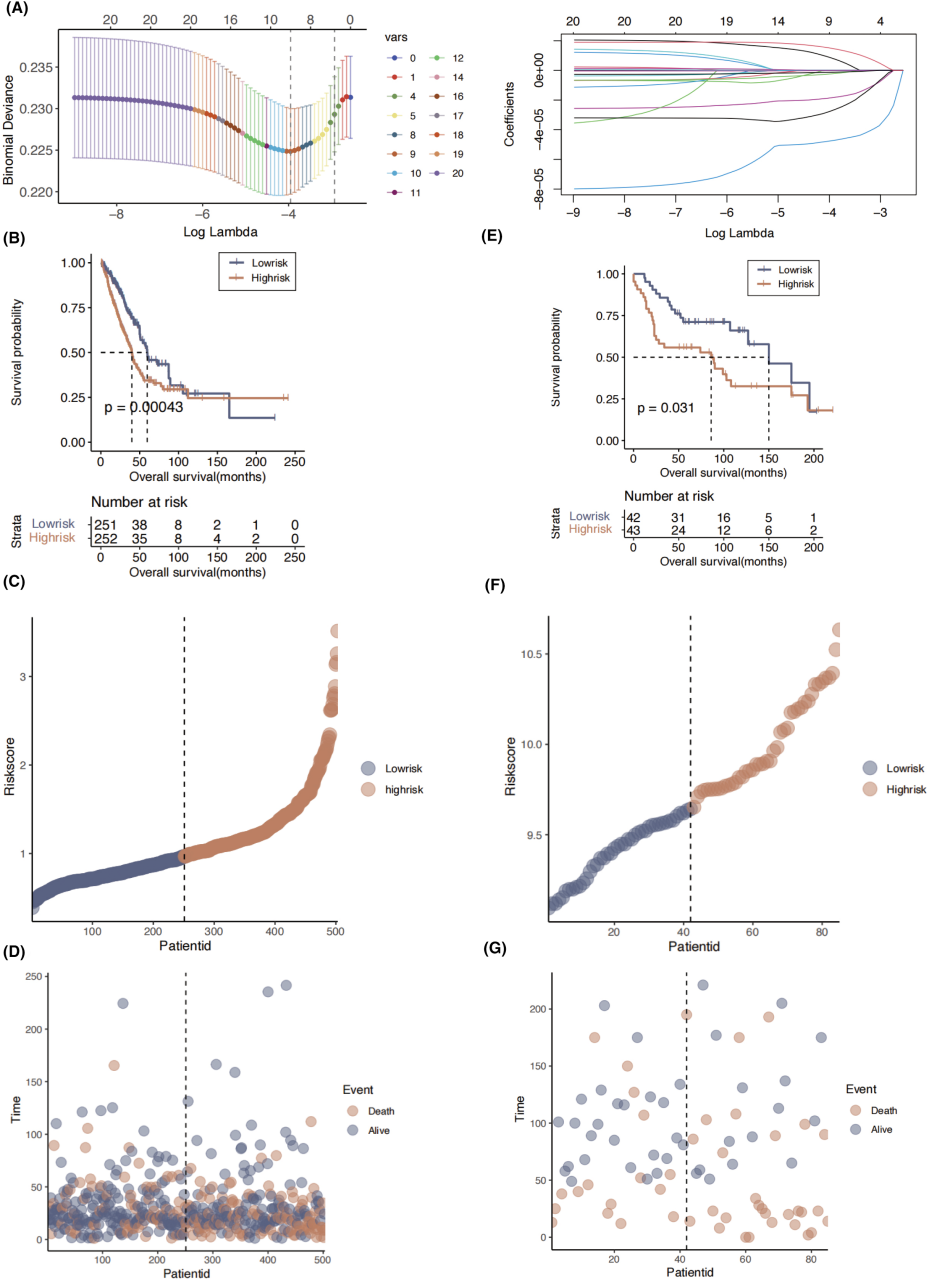
Establishment of risk score model. (A) The parameters of Lasso regression analysis. (B) The survival curve of different risk score groups in training cohort. (C) The risk score value distribution between the low‐risk group and the high‐risk group in training cohort. (D) The survival state and time of patients in the low‐risk group and the high‐risk group in training cohort. (E) The survival curve of different risk score groups in validation cohort. (F) The risk score value distribution between the low‐risk group and the high‐risk group in validation cohort. (G) The survival state and time of patients in the low‐risk group and the high‐risk group in validation cohort.

Univariable COX regression analysis was performed on 125 DEGs between polyamine metabolism‐High and Low groups. Then, 20 genes with *p* < 0.05 were selected. We implemented the LASSO algorithm to further screen out six genes with prognostic significance, namely RORA, CLEC2D, NPC2, A2M, FYN and KLF4 (Figure 4A). The six key genes were utilized for risk score model establishment, which was subsequently used to assessment of the prognosis prediction potency in the training set of TCGA‐LUAD. The risk score calculation is: FYN*0.001 + KLF4*0.174 + A2M*(−0.113) + CLEC2D*(−0.146) + NPC2*(−0.108) + RORA*(−0.055). We observed significant prognostic difference in the TCGA‐LUAD (*p* = 0.00043, Figure 4B–D). Eighty‐five LUAD patients in the GSE30219 dataset were enrolled for external validation, and we also observed significant poor prognosis in the high‐risk group (*p* = 0.031, Figure 4E–G).

**FIGURE 5:**
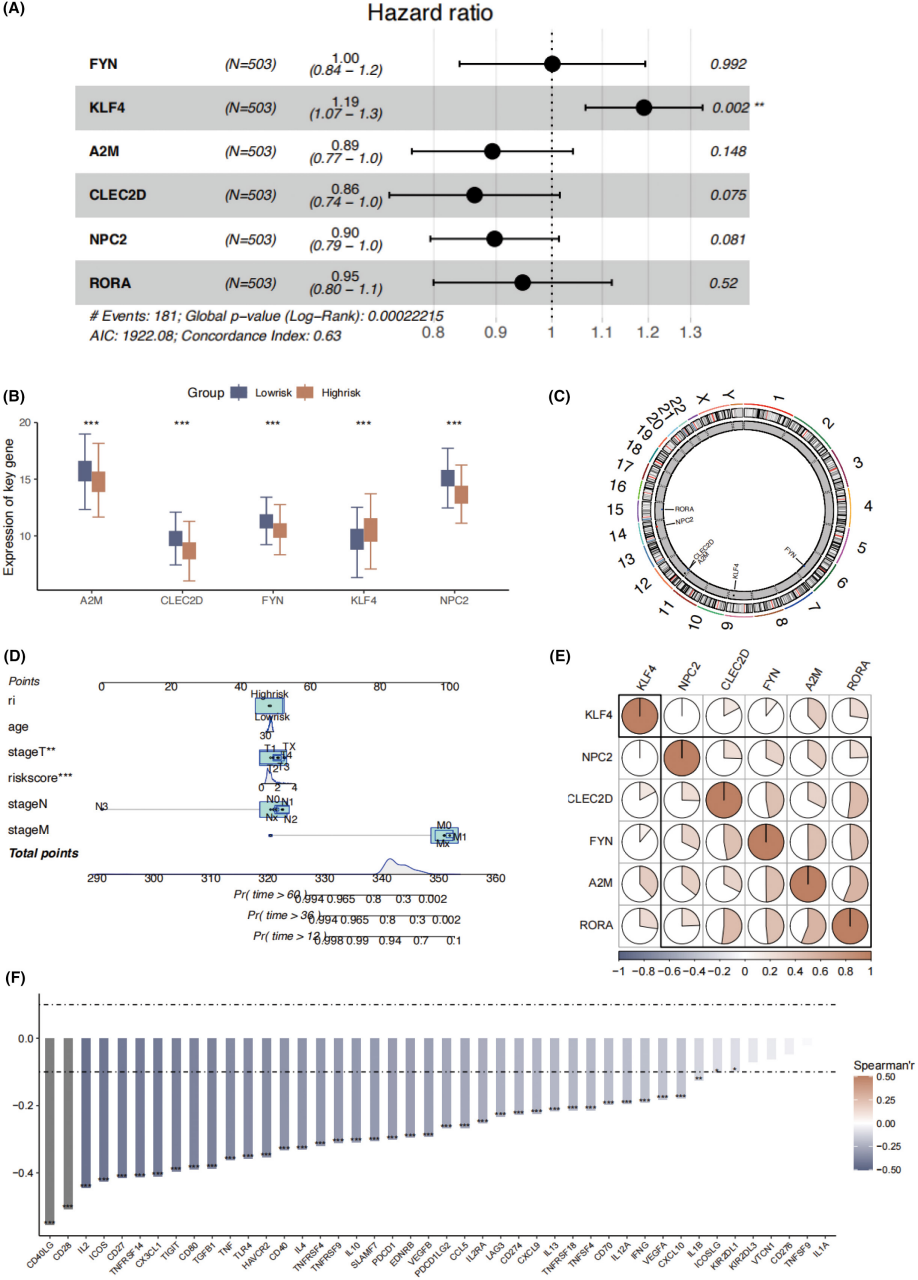
The characterization of the six key genes of risk score. (A) multivariate Cox regression analysis of 6 key genes. (B) The expression levels of six key genes in the LUAD high‐risk and low‐risk groups. (C) Chromosomal distribution of the six key genes. (D) The nomogram integrating pathological stage, age and risk score. (E) The correlation of six key genes. (F) The relationship between the risk score and expression of immunotherapy checkpoint genes. *p < 0.05; **p < 0.01; ***p < 0.001.

We further characterized the 6 key genes by multivariable COX regression analysis. KLF4 was identified as an independent risk factor for LUAD (Figure 5A). We observed significant expression difference across the six selected key genes between two risk groups (Figure 5B). A2M, CLEC2D, FYN and NPC2 were elevated in the low‐risk group, while KLF4 was highly‐expressed in the high‐risk group. The distribution of 6 key genes on the chromosome was displayed in Figure 5C. We further optimized our model into nomogram by integrating clinical factors, including age, pathological stage of T, N, M into the final model for clinical application (Figure 5D). Among the six key genes, we found highest expression correlation between the RORA and CLEC2D, FYN and KLF4 (Figure 5E). Moreover, we conducted a correlation analysis studying the relationships between risk score and the expression levels of common immunotherapy checkpoint genes. We found a universal negative correlation between immunotherapy checkpoint genes and risk score (Figure 5F), suggesting LUAD patients with lower risk score was associated with higher immunotherapy response.

### 3.5 Landscape of immune cells and immune regulating cells as well as mutation analysis in different risk groups of LUAD

**FIGURE 6:**
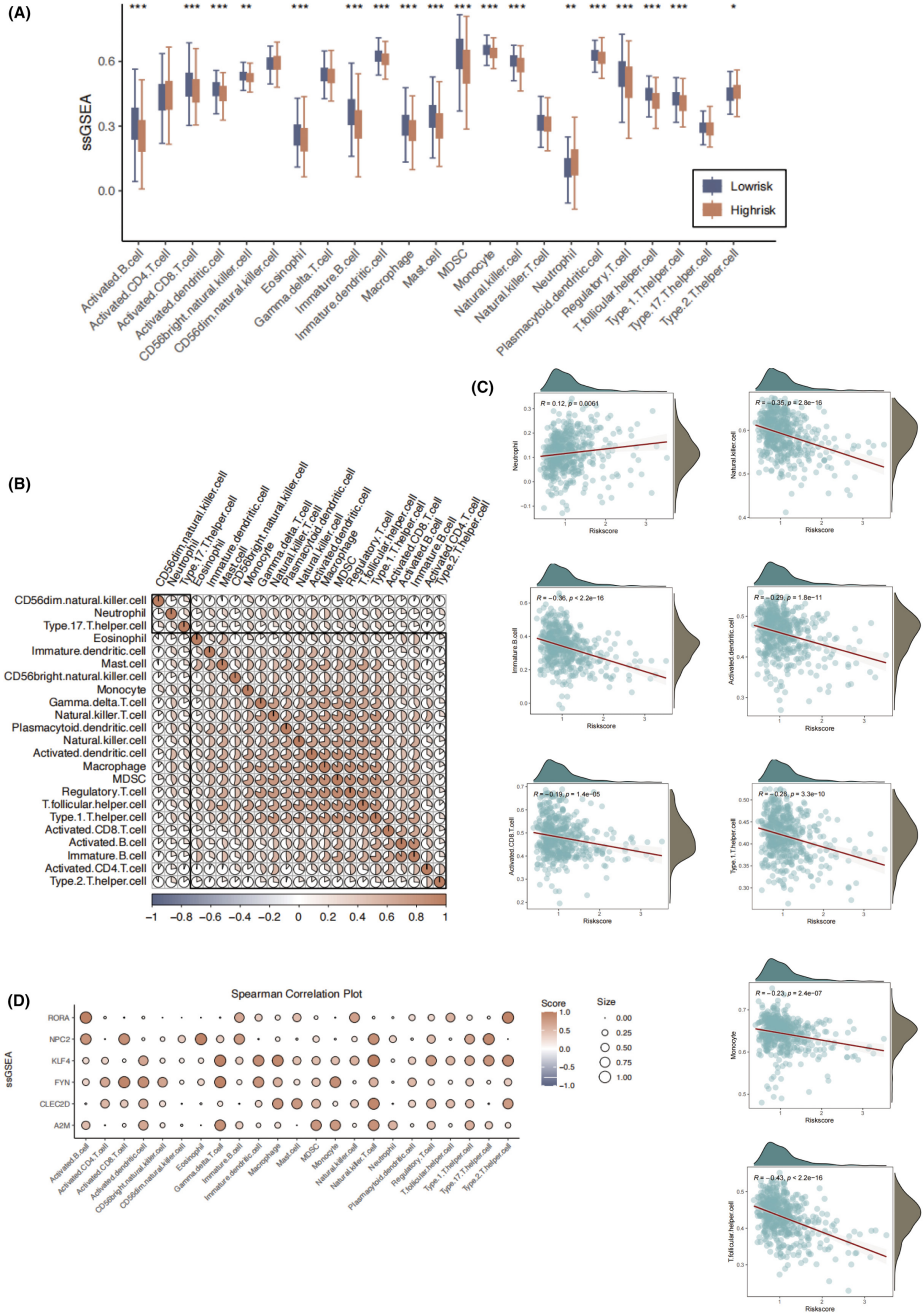
Correlation analysis of immune cell infiltration and risk score. (A) The immune cells and immune regulatory cells infiltration levels based on the ssGSEA algorithm in the high‐risk and low‐risk groups. (B) The correlations between different types of immune cells and immune regulatory cells based on the ssGSEA algorithm. (C) The correlation between risk score and immune cell infiltration. (D) The correlation between the 6 key genes and immune cell and immune regulatory infiltration based on the ssGSEA algorithm. *p < 0.05; **p < 0.01; ***p < 0.001.

We evaluated the status of immune and immune regulating cells infiltration in the different risk score groups. ssGSEA analysis suggested that higher abundance of activated B cells and CD8 T cells in the low‐risk group (Figure 6A). As regard to innate immune cells, we observed that low‐risk group was characterized with higher infiltration of dendritic cells, monocytes, macrophages and mast cells, suggesting a generally activated immune microenvironment in the low‐risk group. The abundance of the MDSC and Tregs was highly correlated with macrophages and activated CD8 T cells (Figure 6B). The neutrophil was positively correlated with risk score value (Figure 6C), which indicated patients with higher risk score might be found to be higher level of intratumor inflammation and necrosis. Consistent with previous analysis, LUAD patients with lower risk score value was associated with elevated immune infiltration of adaptive immune cells, which may partly explained the superior prognosis of low risk group. Among the 6 key genes, FYN expression was highly correlated with functions of cytotoxic T cells, which was featured by activated dendritic cells and CD8 T cells (Figure 6D). KLF4 was mainly associated with higher infiltration of immune suppressive cells. KLF4 was identified as the only independent risk factor for LUAD, which might be largely attributed to its possible contribution to immune suppression in LUAD.

**FIGURE 7:**
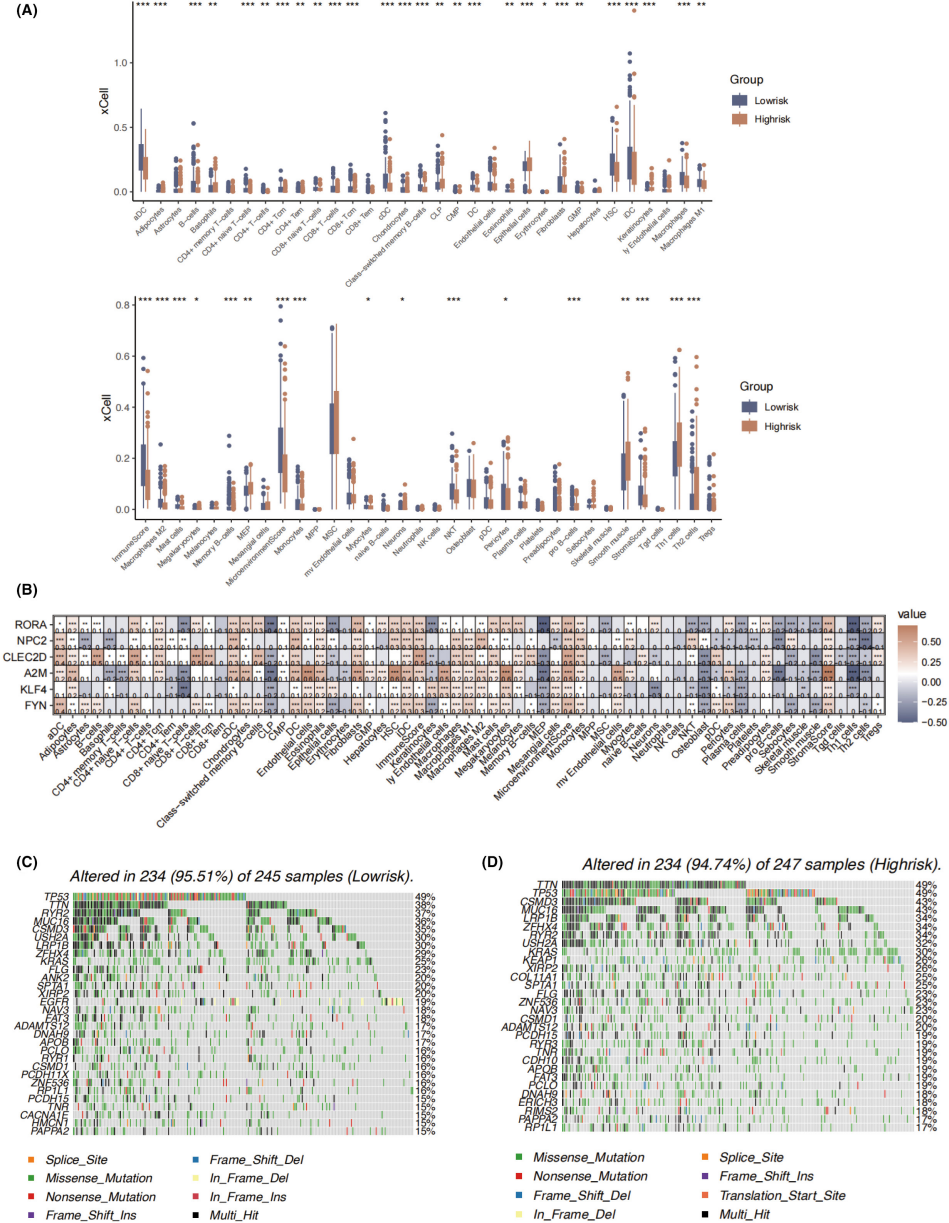
The infiltration of immune and immune regulating cells assessed by xCell algorithm and mutation profiling in the different risk groups. (A) The infiltration of immune cells and immune regulating cells based on xCell algorithm in the high‐risk and low‐risk groups. (B) The correlation between the six key genes and immune cell infiltrates based on xCell algorithm. (C) Mutation type distribution of the top 30 genes in the low‐risk group. (D) Mutation type distribution of the top 30 genes in the high‐risk group. *p < 0.05; **p < 0.01; ***p < 0.001.

We used the xCell algorithm to further evaluate the immune landscape. Consistently, the cytotoxic T cells was elevated in the low‐risk group, along with the fibroblasts, dendritic cells, and macrophages. The immune score was found to be considerably higher than that in low‐risk group (Figure 7A). Correlation analysis revealed that prominent negative correlation between KLF4 and CD8 naïve T cells (Figure 7B).

In mutation analysis, TP53 and TTN accounted for the most frequent mutated genes in both risk groups (Figure 7C,D). The missense mutation was the most prevalent mutation type in both groups. In particular, the mutation rate of TTN in low‐risk group was significantly lower than that of high‐risk group, and the mutation rate of EGFR in low‐risk group was significantly higher than that of high‐risk group.

### 3.6 Drug sensitivity analysis

**FIGURE 8:**
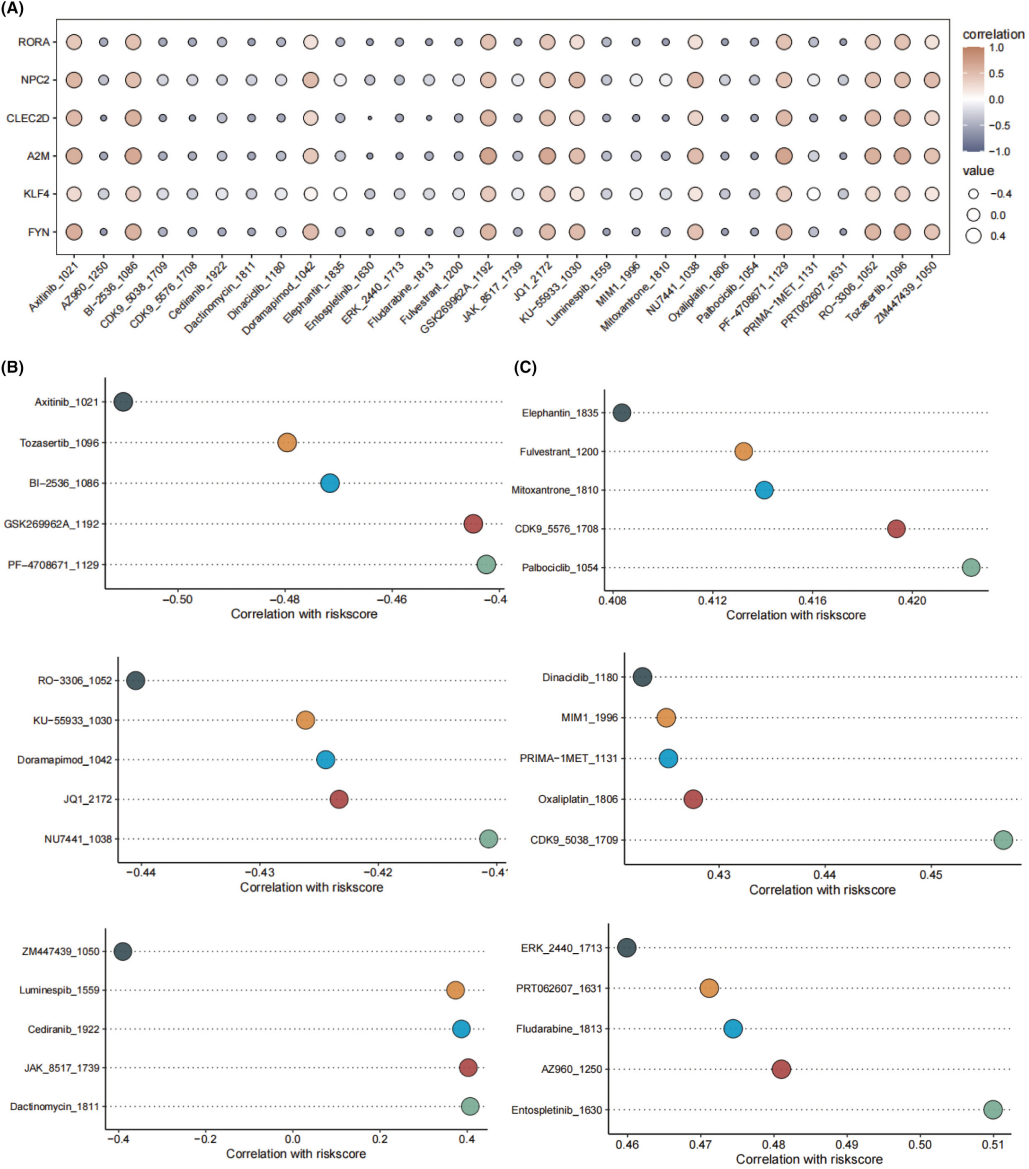
Drug sensitivity analysis of the different risk groups. (A) The correlations between the 6 key genes and drug sensitivity of drugs. (B) The drugs with negative correlations between drug sensitivity scores and risk score. (C) The drugs with positive correlations between drug sensitivity scores and risk score.

We calculated drug sensitivity scores for patients with different risk scores in order to explore potential drugs that could be combined with platinum‐based chemotherapy to improve efficacy (Figure 8A). All six key genes exhibited a similar correlation pattern with several drugs, such as Axitnib_1021, BI‐2536_1086, Doramapimod_1042 and JQ1_2172. In addition, we calculated the association between drug sensitivity score of these drugs and the risk score. Figure 8B shows drugs that are negatively correlated with risk score, such as Axitnib_1021, Tozasertib_1096, and BI‐2536_1086. Figure 8C shows the drugs positively correlated with risk score, such as CDK9_5576_1708 and Palbociclib_1054.

## 4 DISCUSSION

Researchers have explored ways to treat lung cancer by affecting polyamine metabolism since several decades.^12^ DL‐alpha‐difluoromethylornithine (DFMO), a selective inhibitor of polyamine biosynthesis, was found to inhibit the growth of cultured human small cell lung carcinoma implants in nude mice. However, due to insufficient distribution to target cells, the following trails of blocking polyamine biosynthesis showed no notable clinical responses.^13^ Indomethacin, a commonly used non‐steroidal anti‐inflammatory drug, has been found to increase the production of an enzyme called spermidine/spermine‐N1‐acetyltransferase (SSAT), which is encoded by the SAT1 gene. SSAT is responsible for expelling polyamines from the cell. Indomethacin increased the expression of SAT1 and the levels of SSAT in non‐small cell lung cancer (NSCLC) cell lines. In A549 cells, it significantly reduced the levels of putrescine and spermidine. Furthermore, the upstream of the polyamine pathway, such as ornithine and methionine, were found to increase. In A549 cells, the increase in ornithine was associated with several metabolites involved in the urea cycle. Moreover, indomethacin exhibited a synergistic effect with MDL72527, a polyamine oxidase/spermine oxidase inhibitor, in A549 cells.^14^ Collectively, these evidences suggested that polyamine metabolism might be a promising direction for treatment target of LUAD.

Many prognostic models have been developed for LUAD prognosis stratification.^15^, ^16^, ^17^ In our study, we got 10 hub genes associated with both platinum‐based chemotherapy and polyamine metabolism, and found that these hub genes mainly affected signalling transduction pathways. Furthermore, we evaluated the activity of platinum‐based chemotherapy‐related genes and polyamine metabolism based on 10 hub genes in snRNA‐seq data, and found that B cells and mast cells with highest polyamine metabolism activity, followed by the fibroblasts, while NK cells were found with lowest polyamine metabolism activity. DEGs between high polyamine metabolism activity group and low polyamine metabolism activity groups were identified, then 6 key genes with prognostic significance were screened out. Subsequently, the 6 key genes were utilized for risk score model establishment, which showed a good ability of prognostic prediction. We still found a universal negative correlation between immunotherapy checkpoint genes and risk score, and the cytotoxic T cells was elevated in the low‐risk group. In mutation analysis, TP53 and TTN accounted for the most frequent mutated genes in both risk groups, and the missense mutation was the most prevalent mutation type in both groups. In particular, the mutation rate of TTN in low‐risk group was significantly lower than that of high‐risk group, and the mutation rate of EGFR in low‐risk group was significantly higher than that of high‐risk group. We also explored potential drugs for two risk groups that could be combined with platinum‐based chemotherapy to improve efficacy. These results implied that our risk score may offer a better clinical utility over existed prognostic models for LUAD.

Among 6 key genes, KLF4 was identified as an independent risk factor for LUAD, and KLF4 was negatively correlated with CD8 naïve T cells. However, KLF4 was associated with increased infiltration of immune suppressive cells. KLF4, Krüppel‐like transcriptional factor is of great significance in inflammation and malignancy. In lung cancer, most evidences suggested a tumour suppressive role of KLF4 in the cases of NSCLC and small cell lung cancer (SCLC). A decrease of KLF4 was observed in the NSCLC tissues and metastatic tumour tissues located in the trachea and main bronchus. Overexpression of Krüppel‐like factor 4 inhibited the migration, invasion and the mesenchymal‐epithelial transition in various NSCLC cell lines through c‐Jun pathway.^18^ A KLF4/PLAC8 axis was reported to regulate NSCLC progression with KLF4 acting as a suppressor to PLAC8 promoter activity.^19^ Numbl contributed to tumorigenesis by suppressing a “stemness” transcriptional program regulated by KLF4, leading to maintaining a pool of progenitor‐like cells.^20^ In NSCLC, KLF4 could be taken as an tumour suppressor with different types of upstream activator. The deletion of KLF4 induced lung tumour formation in a mouse model. Additionally, analysis of KLF4 promoter methylation and epigenetic factors revealed KLF4 was repressed by histone acetylation.^21^ As a pluripotent transcription factor for cancer stem cells, KLF4 was intimately implicated in cancer drug resistance.^22^ Moreover, a KLF4/p53/miR‐145 regulatory circuit was found in regulating NSCLC drug sensitivity.^23^ In this study, we found KLF4 may be associated with LUAD polyamine metabolism and platinum‐based chemotherapy. However, the effects of KLF4 on functions and mechanisms of LUAD still need to be verified by further cellular experiments.

Our study bears certain limits. We failed to perform valid experiments to further characterize the underlying mechanism regarding how these polyamine metabolism‐associated genes mediate the platinum‐based chemotherapy in LUAD. Moreover, further validation in larger multi‐centre cohorts could verify the efficacy of our model and optimize the model construction.

## 5 CONCLUSION

Collectively, we explored prognostic prediction value of platinum‐based chemotherapy and polyamine metabolism related genes in LUAD by integrating scRNA and bulk RNA‐seq data. We identified six genes (RORA, CLEC2D, NPC2, A2M, FYN and KLF4) to construct a prognostic risk score model, which have potential to prognostic prediction, and could distinguish the characteristics of different immune cell infiltration and gene mutations.

## AUTHOR CONTRIBUTIONS

**Minjun Du:** Resources (equal); software (equal); supervision (equal); validation (equal); visualization (equal); writing – original draft (equal); writing – review and editing (equal). **Xiangzhi Meng:** Resources (equal); software (equal); visualization (equal); writing – original draft (equal); writing – review and editing (equal). **Boxuan Zhou:** Resources (equal); software (equal); writing – original draft (equal); writing – review and editing (equal). **Weijian Song:** Resources (equal); software (equal); validation (equal); writing – original draft (equal); writing – review and editing (equal). **Jianwei Shi:** Resources (equal); software (equal); visualization (equal); writing – original draft (equal); writing – review and editing (equal). **Mei Liang:** Resources (equal); software (equal); supervision (equal); writing – original draft (equal); writing – review and editing (equal). **Yicheng Liang:** Resources (equal); software (equal); supervision (equal); validation (equal); visualization (equal); writing – original draft (equal); writing – review and editing (equal). **Yushun Gao:** Resources (equal); software (equal); supervision (equal); validation (equal); visualization (equal); writing – original draft (equal); writing – review and editing (equal).

## CONFLICT OF INTEREST STATEMENT

The authors confirm that there are no conflicts of interest.

## Data Availability

All datasets generated for this study are included in the article material.
